# A feasibility trial of prehabilitation before oesophagogastric cancer surgery using a multi-component home-based exercise programme: the ChemoFit study

**DOI:** 10.1186/s40814-022-01137-6

**Published:** 2022-08-09

**Authors:** Jakub Chmelo, Alexander W. Phillips, Alastair Greystoke, Sarah J. Charman, Leah Avery, Kate Hallsworth, Jenny Welford, Matthew Cooper, Rhona C. F. Sinclair

**Affiliations:** 1 Northern Oesophago-gastric unit, Royal Victoria Infirmary, Newcastle upon Tyne Hospitals NHS Foundation Trust, Newcastle upon Tyne, NE4 1LP UK; 2 Translational and Clinical Research Institute, Faculty of Medical Sciences, Newcastle University, Newcastle upon Tyne, UK; 3 School of Medical Education, Newcastle University, Newcastle upon Tyne, UK; 4 Northern Centre for Cancer Care, Freeman Hospital, Newcastle upon Tyne Hospitals NHS Foundation Trust, Newcastle upon Tyne, UK; 5 Centre for Rehabilitation, School of Health & Life Sciences, Teesside University, Tees Valley, UK; 6 Department of Anaesthesia and Critical Care, Royal Victoria Infirmary, Newcastle upon Tyne Hospitals NHS Foundation Trust, Newcastle upon Tyne, UK

**Keywords:** Prehabilitation, Oesophagogastric cancer, Feasibility, Surgical oncology, Exercise, Home-based training

## Abstract

**Background:**

Treatment for locally advanced oesophagogastric adenocarcinoma involves neoadjuvant chemotherapy which has a negative impact on patient fitness. Using ‘prehabilitation’ to increase activity levels and fitness may affect physiology, postoperative outcomes and improve patient wellbeing and quality of life. The aims of the trial were to address the feasibility and acceptability of recruiting participants to a home-based prehabilitation programme and provide data to allow design of future studies.

**Methods:**

We recruited patients to a single-arm feasibility trial of home-based exercise prehabilitation. Eligible patients were aged ≥18years, had operable oesophageal or gastric adenocarcinoma and were receiving neoadjuvant chemotherapy at our tertiary referral hospital. All participants commenced a home-based exercise programme utilising pedometers and step counting to target daily aerobic exercise sessions alongside daily strengthening exercises. A weekly telephone consultation directed the exercise programme and facilitated weekly data collection. The primary (feasibility) outcomes for the trial were (a) recruitment rate, (b) completion rate, (c) engagement with the programme (use of pedometers, recording step counts, telephone consultations) and (d) compliance with exercise sessions, exercise intensity and strengthening exercises.

**Results:**

There were 42 patients recruited, and the recruitment rate was 72.4% (42/58). 92.3% (36/39) of patients completed the exercise programme. There was 98.7% (IQR 93.2–100.0%) compliance with wearing a pedometer and recording data, and 100.0% (IQR 93.1–100.0%) compliance with a weekly telephone consultation. Exercise sessions and strengthening exercises were completed 70.2% (IQR 53.1–88.9%) and 69.4% (IQR 52.1–84.3%) of the time, respectively. Appropriate exercise intensity was recorded 96% (IQR 85.4–99.4%) of the time. There were no adverse events. Participants were enrolled in the exercise programme for a median of 91 days (IQR 84 to 105 days).

**Conclusions:**

The results of this trial support the feasibility and acceptability of recruiting participants to an appropriately powered randomised controlled trial of prehabilitation.

**Trial registration:**

Clinicaltrials.gov NCT04194463. Registered on 11th December 2019—retrospectively registered.

**Supplementary Information:**

The online version contains supplementary material available at 10.1186/s40814-022-01137-6.

## Key messages regarding feasibility

- The results of this trial demonstrate that a home-based prehabilitation is safe.
- They also confirm the feasibility and acceptability of the prehabilitation home-based exercise programme.
- The results also confirm the feasibility of recruiting participants to an appropriately powered randomised controlled trial of prehabilitation. The secondary outcome data will enable power calculations to collect these outcomes within a definitive randomised trial.

## Background

Oesophagogastric cancer affects more than 1.4 million people globally each year [1, 2]. For patients with locally advanced oesophagogastric adenocarcinoma, treatment includes a combination of neoadjuvant chemotherapy and surgical resection [3, 4]. This surgery is associated with significant morbidity and mortality [5]. Lower preoperative cardiorespiratory reserve (fitness) is associated with increased postoperative morbidity [6–14]. A sustained reduction in cardiorespiratory reserve occurs during neoadjuvant chemotherapy for oesophagogastric and colorectal cancer [15–17].

Prehabilitation, including preoperative exercise training, can lead to improvements in cardiorespiratory fitness prior to surgery [18–20]. Previous studies have reported improvements in objectively measured cardiorespiratory reserve and health-related quality of life (HRQoL) after supervised in-hospital high-intensity exercise programmes [20–22]. Systematic reviews of prehabilitation before abdominal surgery have demonstrated that the effect of exercise training on postoperative outcomes is poorly reported [23, 24]. However, one recent randomised controlled trial (RCT) reported a 51% reduction in the number of patients who suffered postoperative complications in a group who undertook community prehabilitation (high-intensity interval training [HIIT], nutritional support and lifestyle change support) before major abdominal surgeries [25].

The best methods of increasing physical fitness prior to major surgery remain unclear. Previous research programmes have used HIIT in-hospital [20–22], community-based exercise [26] or home-based exercise programmes [27]. Home-based exercise with remote supervision presents an attractive option with potential advantages of being scalable, accessible and reducing the burden of delivery within the hospital system. There is conflicting evidence on patient preferences regarding the types of fitness training, the structure and the location of the exercise programme [28–30]. The heterogenous nature of previous exercise regimens supports the notion that ‘one size does not fit all’ and that multiple factors will determine the preferred exercise programme for different groups of patients. Importantly, a ‘personalised’ regimen needs to be deliverable, acceptable and achieve the desired outcomes. We hypothesise that a home-based exercise intervention will be an appropriate and achievable way of improving cardiorespiratory fitness before major surgery for our oesophagogastric cancer patients who have varied levels of baseline fitness, need to maintain fitness during preoperative chemotherapy and come from a wide geographical area to our tertiary referral centre.

Feasibility of prehabilitation has been previously demonstrated; however, there is a large degree of heterogeneity amongst the exercise regimens and target populations. There is a little known on whether home-based prehabilitation with remote support for patients with oesophagogastric cancer during neoadjuvant chemotherapy is feasible.

We performed this study to address the feasibility and acceptability of recruiting participants to a home-based prehabilitation exercise programme prior to oesophagogastric cancer surgery. The primary objectives of the trial were as follows:

1. Assess whether patients would agree to take part in a home-based exercise programme during treatment for oesophageal or gastric cancer.
2. Assess whether the participants would engage with the program and continue with it throughout their cancer treatment and therefore determine its acceptability to the participants.
3. Assess whether participants would utilise the tools and structure of the programme we had conceived.

The secondary objectives were as follows:

1. Ensure that we could measure outcomes that would be required in a fully powered study of the intervention.
2. Provide data to allow power calculation for a full randomised study.

## Methods

This was a prospective, single-centre, feasibility study conducted between February 2019 and March 2020. Participants were identified during the multidisciplinary cancer staging process and included patients with locally advanced oesophageal and gastric adenocarcinoma receiving neoadjuvant chemotherapy [31]. Exclusion criteria included patients <18 years old, contraindications to cardiopulmonary exercise testing [32], or patients who were not deemed suitable for neoadjuvant chemotherapy. All patients who met the inclusion criteria during screening at the multidisciplinary meeting were approached to discuss participation in the study and were provided with a patient information sheet. We recorded the reasons reported by patients who declined to participate. Informed written consent was taken from all patients recruited to the study.

The Health Research Authority (REC 18/WA/0427) provided ethical approval for this study. The study was registered at ClinicalTrials.gov (NCT04194463). All procedures performed were in accordance with the Declaration of Helsinki.

### Intervention: home-based exercise programme

**Fig. 1:**
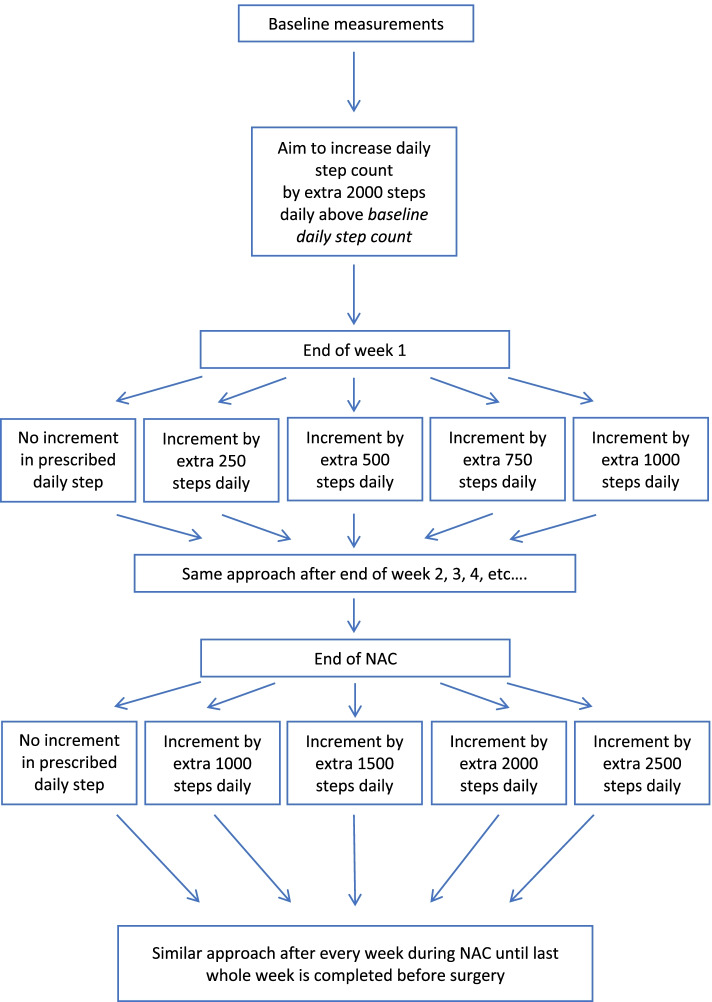
The structure of the exercise programme

The home-based exercise programme consisted of a combination of targeted daily step-based aerobic exercise and daily strengthening exercises as per protocol [31]. To summarise, participants were provided with a pedometer (Walking style One 2.1, Omron Healthcare UK Ltd., UK), resistance band (BodyMax resistance tube, BodyMax Ltd., UK) and exercise diaries. Each week continued engagement with the programme was reinforced during a weekly phone call by a researcher who provided motivational discussions and collected data on daily step count. Participants were involved in the programme during neoadjuvant chemotherapy and continued involvement after this finished until a week before surgery. The exercise intervention aimed to achieve an increase in daily step count of 2000 steps above baseline level, 7 days per week. The structure of the exercise programme is outlined in Fig. 1. The exercise regimen and strengthening exercises are described in detail in supplementary content 1 and in the published protocol [31].

### Interim review of step increments

A planned interim review was undertaken after the first 10 participants had commenced exercise [31]. At interim review, eight of the ten participants had not achieved their prescribed daily step count of 2000 steps per day above the baseline daily step count for at least five days of the first week of exercise. This rate of ‘failure to achieve the prescription’ exceeded our predefined specifications for adapting the protocol. The exercise protocol was subsequently amended and the increment of daily steps was reduced to a target of 1000 steps per day above the baseline daily step count for all participants recruited thereafter.

*The feasibility outcomes (primary outcomes) for the study were as follows:*

1. Recruitment rate: The proportion of all patients approached that agreed to enter the study.
2. Completion rate: The proportion of all participants who entered the study and remained in the study at the end of the defined study period.
3. Engagement with the programme: the percentage of intervention days that each participant wore the pedometer and was recording step count data.
4. Completed telephone consultations: the percentage of weekly telephone consultations that were completed.
5. Compliance with each daily aerobic session: the percentage of intervention days achieved.
6. Compliance with achieving target intensity (rate of perceived exertion [RPE] on Borg scale) [33]: the percentage of aerobic sessions that reached target intensity.
7. Compliance with daily strengthening sessions: the percentage of intervention days achieved.

Compliance outcomes were obtained by the research team during telephone contacts with participants who were instructed to complete their exercise diaries on daily basis. The secondary outcomes are detailed in the protocol [31] and included reported daily step count at each time point during the study, measurement of cardiopulmonary fitness before and after the home-based exercise programme, sarcopenia measured by computed tomography (CT) scan analysis [34, 35] and hand grip strength using a dynamometer, and quality of life using EORTC QLQ-C30 tool [36].

### Statistical analysis

A recruitment target of 40 patients was selected in accordance with published guidance for feasibility studies [37, 38]. This sample size was felt to be sufficient to inform feasibility of recruitment and retention to the study and to examine the practicalities of the intervention.

An interim analysis of step count data was planned (see above). Continuous data were described as mean ± SD if normally distributed and median (IQR) if not using the Shapiro-Wilk test to test the assumptions of normality. Categorical data were described using frequencies and compared using chi-squared test. Comparison between pre- and post-intervention continuous data was made using Student’s *t* paired test. The effect of intervention was estimated using 75% confidence intervals of mean difference. All analyses were performed using IBM® SPSS® Statistics version 26 (IBM, Armonk, USA).

## Results

**Fig. 2:**
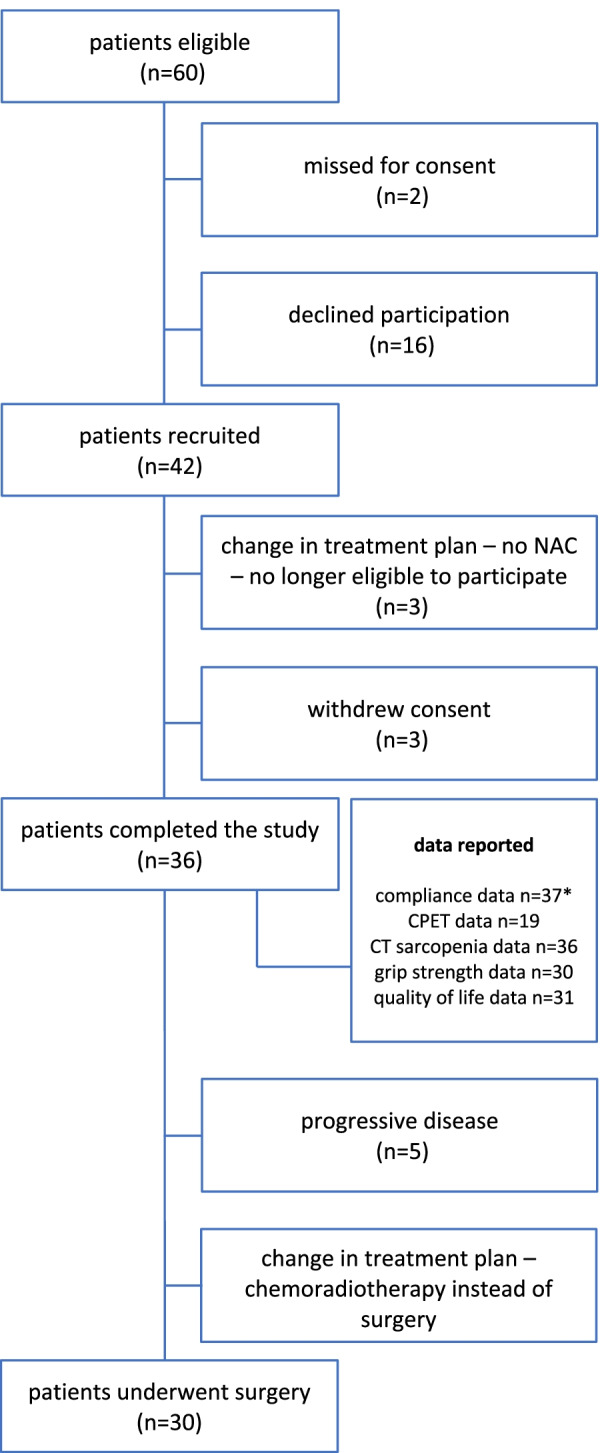
The flow of participants from screening onwards

Forty-two patients were recruited to this feasibility trial and 36 completed the study. Figure 2 shows the flow of participants from screening onwards. There were 60 patients who were identified as eligible to enter the trial. We missed the opportunity to take consent from two of these patients and a further 16 chose not to participate in the trial.

The participants’ clinical characteristics are presented in Table 1. The median age was 68 years old (range 51 to 81 years) and 33 (85%) participants were male. Participants were enrolled in the exercise programme for a median of 91 days (IQR 84 to 105 days). A median of 45 days (IQR 44 to 63 days) was spent receiving chemotherapy. The median duration of exercise programme completed after cessation of chemotherapy and before surgery was 35 days (IQR 31 to 47 days).

**Table 1:** Characteristics of all enrolled patients (including patients who later withdrew consent)

	39 participants
Age (years), median (range)	68 (51 to 81)
Gender, male, *n* (%)	33 (85)
BMI (kg.m^-2^), median (range)	27.3 (19.7 to 41.3)
Smoking status	
Never	9
Ex-smoker >1 year	24
Current smoker	6
Comorbidities	
Asthma/COPD	8
Diabetes mellitus	5
Ischaemic heart disease	1
Atrial fibrillation	2
Cerebrovascular disease	1

*Abbreviations*: *BMI* body mass index, *COPD* chronic obstructive pulmonary disease

### Feasibility results

The recruitment rate was 72.4% (42/58 patients approached). The reasons reported for non-participation (16 patients) were nine patients did not want to travel to our centre for baseline measurements/consent/introduction to the programme, three patients felt that they were ‘fit enough already’, two patients stated that they had too many appointments already, one patient felt ‘too weak’ and one patient ‘did not want to’ participate. Three patients had a change to their treatment plan shortly after being recruited which meant they did not receive neoadjuvant treatment: they no longer met the inclusion criteria and were excluded. Thirty-nine participants commenced the home-based exercise programme, and the completion rate was 92.3% (36/39). Three participants withdrew from the study. Two of these participants found the regimen to be very demanding in conjunction with their treatment. One participant found regimen to be very time consuming. There were no adverse events related to the intervention. The primary outcomes are summarised in Table 2.

**Table 2:** Feasibility outcomes

Recruitment rate, *n* (%)	42/58 (72.4%)
Completion rate, *n* (%)	36/39 (92.3%)
Compliance with the regimen (wearing pedometer and recording data), median % IQR	97.8% [93.2 to 100.0%]
Completed telephone consultations, median % IQR	100.0% [93.1 to 100.0%]
Compliance with a daily aerobic exercise session, median % IQR	70.2% [53.1 to 88.9%]
Compliance with achieving target intensity (RPE Borg), median % IQR	96.7% [85.4 to 99.4%]
Compliance with completion of daily strengthening sessions, median % IQR	69.4% [52.1 to 84.3%]

Compliance with the aerobic session was 64.8 versus 71.8% and with strengthening exercises was 69.7 vs 68.6%, during and after chemotherapy, respectively.

### Secondary outcomes

#### Pedometer data

Pedometer data was recorded on 3284 of 3473 possible participant days (94.6%). The range of daily step counts reported was wide (0–26,533). The mean daily step count also showed variation when calculated for different time intervals—at baseline, during chemotherapy (NAC) and after chemotherapy whilst awaiting surgery (post-NAC). During the baseline period this was 5528 [IQR 2303 to 8515] steps/day which decreased during NAC to 5121 [IQR 2512 to 7712] steps/day and increased post-NAC to 5792 [IQR 2361 to 9980] steps/day.

#### Cardiopulmonary exercise tests

Thirty-six participants completed baseline cardiopulmonary exercise test (CPET) and 19 completed the end of study CPET test because the trial was disrupted by the COVID-19 pandemic which forced the closure of our CPET facility in March 2020 to ensure patient safety [39]. A comparison of baseline vs post-exercise CPET measurements is only available for 19 participants (Table 3). Mean baseline oxygen uptake at anaerobic threshold (VO_2_ at AT) and mean baseline peak oxygen uptake (VO_2_peak) were 14.3ml.min^-1^.kg^-1^ and 19.4ml.min^-1^.kg^-1^, respectively. End of study CPET demonstrated VO_2_ at AT of 13.9ml.min^-1^.kg^-1^ and VO_2_peak of 19.3ml.min^-1^.kg^-1^.

**Table 3:** Comparison of mean ± SD cardiopulmonary exercise testing measurements at baseline and after exercise regimen

	Baseline CPET (*n*=19)	End of study CPET (*n*=19)	75% CI
VO_2_ at AT (ml.min^-1^.kg^-1^)	14.3 ± 3.2	13.9 ± 2.8	−0.2;1.1
VO_2_peak (ml.min^-1^.kg^-1^)	19.4 ± 4.2	19.3 ± 4.2	−0.7;0.9
VE/VCO_2_	30.6 ± 3.8	31.5 ± 4.3	−1.5; −0.3
FEV1 (l)	2.6 ± 0.8	2.6 ± 0.7	−0.1;0.1
FVC (l)	3.6 ± 0.9	3.6 ± 0.9	−0.1;0.1

*Abbreviations*: *VO*_*2*_ *at AT* oxygen uptake at anaerobic threshold, *VO*_*2*_*peak* peak oxygen uptake, *VE/VCO*_*2*_ ventilatory equivalents for carbon dioxide, *FEV1* forced expiratory volume in 1s, *FVC* forced vital capacity, *CPET* cardiopulmonary exercise testing, *CI* confidence interval

#### Sarcopenia

CT sarcopenia was measured at baseline and at the end of the study in 36 participants. A decline in muscle bulk at the L3 level was seen, mean lean body mass changed from 52.3 to 49.1kg. There was an increase in the proportion of the participants who met the threshold for CT defined sarcopenia (from 47 to 72%). There was no change in grip strength. This was 34.4kg at the baseline compared to 33.6kg at the end of the study (Table 4).

**Table 4:** Sarcopenia measurements

	Baseline	End of study	75% CI
CT defined sarcopenia present, *n* (%)^a^	17 (47.2%)	26 (72.2%)	N/A
Lean Body Mass, kg^a^	52.3 ± 9.8	49.1 ± 9.4	−3.8; −2.5
Grip strength, kg^b^	34.4 ± 8.8	33.6 ± 9.0	−2.6; 1.0

*Abbreviations*: *CT* computed tomography, *CI* confidence interval

^a^Analysed on 36 participants. ^b^Analysed on 30 participants

#### Quality of Life

Quality of life questionnaires were completed by 39 and 31 participants at baseline and at the end of the study, respectively. The mean score of global health status improved from 65.32 to 78.23. Participants reported an improvement in physical function and decreased fatigue. Improvements were also reported in nausea and appetite (Table 5).

**Table 5:** Quality of life scores, mean ± SD at baseline, and at the end of the study

	Baseline	End of study	75% CI
**Global health status/QoL (QL2)**	65.32 ± 17.76	78.23 ± 21.16	9.0; 16.8
**Functional scales**			
Physical functioning (PF2)	85.59 ± 18.39	91.40 ± 15.98	3.0; 8.6
Role functioning (RF2)	84.41 ± 26.85	86.02 ± 24.76	−3.5; 6.7
Emotional functioning (EF)	74.73 ± 23.12	79.03 ± 23.06	−1.1; 9.7
Cognitive functioning (CF)	89.78 ± 19.09	91.40 ± 15.44	−2.1; 5.3
Social functioning (SF)	79.57 ± 28.45	82.80 ± 23.76	−1.8; 8.2
**Symptom Scales**			
Fatigue (FA)	28.49 ± 24.20	21.86 ± 20.79	−10.0; −3.3
Nausea (NV)	15.59 ± 18.73	5.38 ± 10.88	−14.0; −6.4
Pain (PA)	14.52 ± 19.60	13.44 ± 24.50	−5.4; 3.3
Dyspnoea (DY)	17.20 ± 20.85	15.05 ± 22.51	−5.7; 1.4
Insomnia (SL)	38.89 ± 31.66	32.26 ± 34.94	−12.4; −0.9
Appetite loss (AP)	38.89 ± 34.00	22.58 ± 36.91	−22.3; −8.9
Constipation (CO)	13.98 ± 25.49	18.28 ± 27.00	−3.5; 12.2
Diarrhoea (DI)	9.68 ± 23.08	6.45 ± 15.91	−9.3; 2.9
Financial difficulties (FI)	18.28 ± 30.84	15.05 ± 27.00	−10.1; 3.6

*Abbreviations*: *CI* confidence interval

## Discussion

This trial has examined the feasibility of recruiting oesophageal and gastric cancer patients to a home-based exercise programme. Thirty-six (92.3%) of the participants who entered the trial completed 70.2% of the daily aerobic exercise sessions and 69.4% of strengthening exercises at home (without attending hospital). The program was safe and feasible demonstrating high compliance to the protocol and low dropout. There were no adverse events. The rates of compliance should be considered in the context of the clinical disease process, including treatment with chemotherapy, its’ potential complications and morbidity.

The recruitment rate in this trial was 72% which is lower than in previous studies of pre-operative exercise interventions in surgical cancer populations which have reported varied recruitment rates ranging from 80% [40] to over 90% [41, 42].

The principal reason reported for not entering the study was the requirement to travel to attend an introductory session, baseline measurements and consent. Travel is reported in other studies as a barrier to participation [28]. In a future definitive trial, this barrier to recruitment should be recognised and alternative means of introducing the study exercise regimen and taking consent should be utilised. This could involve remote consultation, online information, online consent or a home visit for consultation and consent. Alternatively, a change in the timing of the consent process to allow flexibility of this within the clinical pathway may increase recruitment (two patients did not participate due to the high volume of appointments). Consideration should be given to participant payments to cover travel costs and inconvenience.

The completion rate we report is consistent with previous reports [41–43]. It is inevitable that during oncological treatment, the clinical treatment strategies can, and do, change to reflect disease progression and patient response to treatment. This can result in non-operative treatment for some patients where surgery had previously been expected with a resultant impact on trial retention rates. During this trial, three patients changed treatment plans which then excluded them from the study shortly after consent, and a further five participants developed progressive disease such that they were no longer appropriate for curative surgery at the end of the study. In a future trial, this will affect both the recruitment strategy and the attrition rate that is used within a power calculation.

The methods of measuring compliance used in previous prehabilitation studies are heterogeneous and compliance rates have varied widely (16–98%) [44–46]. The compliance rates in this trial were high. The UK-based PREPARE regimen employed similar targets for preoperative exercise, including targeted 30-min walks, 5 times per week as one of its patient targets [41]. This group have successfully enrolled 67 oesophagogastric surgical patients (2016–2018) and report weekly adherence as an average of 64% of sessions completed [41]. Their weekly adherence was lower during chemotherapy (56%) and increased after chemotherapy during the time before surgery [41]. This is in contrast to our feasibility outcomes where compliance was not different at each of the before, during or after chemotherapy time points. Many features common to previous home-based prehabilitation schemes are included in this trial [41, 42]: patient-directed goal setting, weekly telephone support, exercise diaries and exercise intensity targets. Goal-setting and weekly telephone calls have contributed to good compliance in this study (median 100% compliance with weekly telephone call) and previous studies [28, 41, 47].

An important area for consideration in this trial was the feasibility of using a pedometer to encourage a step-based exercise regimen and weekly goal setting. Whilst different home-based exercise programmes using remote supervision have previously been reported [10, 27, 44, 48], these programmes have not used step counting or pedometers as the driver of targeted activity. We have found that participants were highly compliant with using a pedometer and recording step counts (97.8% compliance). Step-counting has become part of the modern exercise armamentarium due to the availability of devices. The concept is simple, and improvement is easily visible to the patient making this an attractive method of motivation and measurement when supervising patients remotely. The advantages of this model include avoiding costly equipment, allowing the patient to exercise at their convenience and facilitating exercise in surroundings that are geographically convenient (near home). The compliance data supports the future use of this methodology, allowing the home-based exercise programme to be accessible to all future patients.

### Limitations

There are limitations within this trial. Selection bias was minimised by assessing all consecutive patients for eligibility with only two eligible patients missed; however, there may have been bias inherent in the patients that declined to take part in the study. This may be overcome in a future study by changing the methodology for consenting to the study and introducing the exercise programme, to avoid unnecessary hospital visits and travel. The trial was not powered to detect differences in secondary outcomes or to compare the prehabilitation programme data with previous results [49]. Our study was interrupted by the COVID-19 pandemic, and despite this, we completed patient recruitment to the study. The pandemic prevented completion of CPET after 23rd March 2020, which has resulted in incomplete secondary outcome data for 17 participants. However, with regard to feasibility, we believe that it is possible to take these measurements at both study time-points and to collect this data (in the absence of further pandemics that affect this service). There remains sufficient secondary outcome data to enable planning of appropriately powered analyses in the future. The CPET facility is now assessing patients again in COVID-safe environment that will continue for the foreseeable future. The measurement of the exercise, step count and Borg RPE score were self-reported by patients into exercise diaries. Although there might be some concerns about validity/truthfulness of the self-reported data (reporting error), patients frequently reported when they did not complete exercise, especially during difficult weeks of chemotherapy. Finally, data about the socio-economic status of participants were not collected, and therefore, their health literacy and consequently likelihood of them being able to complete the exercise programme cannot be evaluated.

The results of this trial confirm the feasibility and acceptability of recruiting participants to an appropriately powered randomised controlled trial of prehabilitation. The recruitment rate supports the ability to recruit to a larger trial, and the compliance rates support the acceptability of the current exercise programme. The secondary outcome data will enable power calculations to collect these outcomes within a definitive randomised trial. Implementation of the prehabilitation regimen used within this study into routine clinical practice would be highly desirable should a future RCT demonstrate that this programme leads to improvement of preoperative fitness and better postoperative outcomes.

## Supplementary Information

**Additional file 1.** Home Exercise Programme.

## Data Availability

The datasets used and analysed during the current study might be available from the corresponding author on reasonable request.

## Abbreviations

HRQoL: Health-related quality of life
RCT: Randomised controlled trial
HIIT: High-intensity interval training
RPE: Rating of perceived exertion
CT: Computed tomography
SD: Standard deviation
IQR: Interquartile range
NAC: Neoadjuvant chemotherapy
post-NAC: Post-neoadjuvant chemotherapy
CPET: Cardiopulmonary exercise test
