# Supplementary material for: A feasibility trial of prehabilitation before oesophagogastric cancer surgery using a multi-component home-based exercise programme: the ChemoFit study
Source: Pilot Feasibility Stud. 2022 Aug 9;8:173. doi: 10.1186/s40814-022-01137-6 (PMC9360697; doi:10.1186/s40814-022-01137-6)
Supplement: Supplementary file 1 — Additional file 1. Home Exercise Programme. [file 40814_2022_1137_MOESM1_ESM.docx]

## Supplementary Content. Home Exercise Programme.

Daily Step Based Programme.

Patients were provided with a pedometer, resistance band and exercise diaries. A full description of the exercise programme was provided by the researcher at the start of the programme. This was reinforced during a weekly phone call by the researcher to the patient which involved motivational discussions and data collection of daily pedometer steps.

For the first week of the exercise programme, the patient’s baseline habitual daily step count was recorded using the pedometer. The targeted exercise programme then commenced and the baseline step count was used to create a new target step count for the participant to aim to complete every day over each forthcoming week. The exercise programme continued and was guided by the patient, throughout the time between recruitment to the study and until surgery. The duration of the programme was variable depending upon the clinical course of the patients’ usual clinical care: the last study visit, and measurements were completed within the two weeks prior to surgery.

The home exercise programme initially involved a prescription to increase daily step count by 2000 steps per day, seven days per week above the baseline 7-day measurement (Figure 1). The initial step increment prescription was based upon a recent study called Active-at-Home HF, which utilised pedometer-based step targets in patients with stable, chronic heart failure.^50^ Patients were asked to complete the prescribed daily steps by walking/jogging at a moderate intensity using the descriptions from the Borg rating of perceived exertion (RPE) 0-10 scale ^33^ and to achieve a RPE level of between 3 and 4. The aim was to achieve the additional steps each day by completing 30 minutes of aerobic exercise.

Strengthening Exercises

In addition to daily walking patients were asked to perform two sets of strengthening exercises each day (each containing five resistance exercises) to target major muscle groups.^31^ The patients were encouraged to perform other physical activities if they were able to do so. The completed daily exercise was recorded by our patients in exercise diaries.

Telephone Support

Each week the patient received a telephone call from the research team, which was designed to motivate the patient, collect the previous weeks’ step data and to set the step (exercise) target for the next week of the programme. The target step count was increased each week during the study period if both the patient and research team felt this was achievable. The research team considered ‘wellness’ or ‘illness’ from the ongoing cancer care and chemotherapy. The content of the intervention and associated support were personalised to each individual and informed by the baseline level of physical activity, age, general health, motivation and social circumstances, with the view to achieve the greatest improvement in cardiopulmonary fitness. The adaptive and flexible nature of our programme was chosen to promote patient control of exercise and to reflect the varied responses and abilities of patients to exercise that we had observed previously in our clinical practice.

After completion of chemotherapy

Once patients had entered a post-chemotherapy phase and were awaiting surgical resection, we requested that they increase their daily step count in the exercise prescription in order to use this time to further improve cardiopulmonary fitness (Figure 1). This was done in discussion with the patient, and again, taking into account of progress with the clinical cancer care.
